# A De-Novo Genome Analysis Pipeline (DeNoGAP) for large-scale comparative prokaryotic genomics studies

**DOI:** 10.1186/s12859-016-1142-2

**Published:** 2016-06-30

**Authors:** Shalabh Thakur, David S. Guttman

**Affiliations:** Department of Cell & Systems Biology, University of Toronto, Toronto, ON Canada; Centre for the Analysis of Genome Evolution and Function, University of Toronto, Toronto, ON Canada

**Keywords:** Comparative genomics, Prokaryotes, Gene prediction, Gene annotation, Ortholog identification, Functional annotation, Pan genome, Core genome, Flexible genome

## Abstract

**Background:**

Comparative analysis of whole genome sequence data from closely related prokaryotic species or strains is becoming an increasingly important and accessible approach for addressing both fundamental and applied biological questions. While there are number of excellent tools developed for performing this task, most scale poorly when faced with hundreds of genome sequences, and many require extensive manual curation.

**Results:**

We have developed a de-novo genome analysis pipeline (DeNoGAP) for the automated, iterative and high-throughput analysis of data from comparative genomics projects involving hundreds of whole genome sequences. The pipeline is designed to perform reference-assisted and de novo gene prediction, homolog protein family assignment, ortholog prediction, functional annotation, and pan-genome analysis using a range of proven tools and databases. While most existing methods scale quadratically with the number of genomes since they rely on pairwise comparisons among predicted protein sequences, DeNoGAP scales linearly since the homology assignment is based on iteratively refined hidden Markov models. This iterative clustering strategy enables DeNoGAP to handle a very large number of genomes using minimal computational resources. Moreover, the modular structure of the pipeline permits easy updates as new analysis programs become available.

**Conclusion:**

DeNoGAP integrates bioinformatics tools and databases for comparative analysis of a large number of genomes. The pipeline offers tools and algorithms for annotation and analysis of completed and draft genome sequences. The pipeline is developed using Perl, BioPerl and SQLite on Ubuntu Linux version 12.04 LTS. Currently, the software package accompanies script for automated installation of necessary external programs on Ubuntu Linux; however, the pipeline should be also compatible with other Linux and Unix systems after necessary external programs are installed. DeNoGAP is freely available at https://sourceforge.net/projects/denogap/.

**Electronic supplementary material:**

The online version of this article (doi:10.1186/s12859-016-1142-2) contains supplementary material, which is available to authorized users.

## Background

Advances in next-generation sequencing technology have revolutionized the field of comparative genomics and enabled researchers to gain much greater resolution and insight into questions related to genome plasticity, molecular epidemiology, and evolution and diversity among closely related species and strains [1–5]. A wide range of powerful tools have been developed to help researchers perform whole genome comparisons; however, it is often difficult to automate these analyses [6–8]. The problem is exacerbated when dealing with draft genomes, since predictive and comparative analyses are often not designed to work with fragmented genes that arise due to sequencing or assembly errors [9]. Consequently, it is usually prudent to use multiple methods that employ different underlying algorithms to minimize the occurrence of false positive or negative results due to algorithm bias or sequencing and assembly errors [10]. While using multiple approaches enhances robustness, it also introduces another set of problems related to the integration of tools that more often than not rely on disparate data formats and structures.

Perhaps the biggest challenge faced during comparative genomic analysis is that most analysis approaches do not scale well when faced with hundreds of genomes. There is very high computational complexity associated with the management and analysis of large genomic datasets. The majority of comparative analytical approaches rely on pairwise sequence comparisons, which result in a quadratic relationship between the number of genomes analyzed and the computational time [11–13]. Such computational complexity is often a bottleneck for large-scale genome analysis projects [14]. It is also becoming increasingly impractical to reanalyze an entire genome database every time new strains are added. As these databases expand to include thousands of strains researchers will need the ability to iteratively add new genomes without reanalyzing the entire existing collection.

Given these challenges to large-scale comparative genomic analysis, we reasoned that a new approach might be needed that can reduce the complexity of automated prediction and annotation, streamline the analysis of large numbers of draft whole genome sequences, and permit iterative analysis. To achieve these goals, we developed the de-novo genome analysis pipeline (DeNoGAP), which integrates existing tools for prokaryotic gene prediction, homology prediction, and functional annotation for both intraspecific and interspecific genome comparison. Importantly, it employs an iterative clustering method to identify homologs and novel gene families using hidden Markov models. The iterative clustering process dramatically reduces the computational complexity of large-scale genome comparisons. DeNoGAP also creates SQLite databases to store analyzed genomic information and provides a graphical interface explorer for browsing and comparison of the predicted information between multiple genomes. DeNoGAP provides a modular architecture that will allow researchers to perform large-scale comparative analysis, generate and test the hypothesis, and create a well-annotated genome database for data analysis and exploration.

## Implementation

### Pipeline organization

DeNoGAP is a command line tool built using Perl scripting language for analysis of complete and draft prokaryotic genome sequences. The pipeline performs four primary analysis tasks: gene prediction, functional annotation, ortholog prediction, and pan-genome analysis. DeNoGAP works for both intraspecific (single species) and interspecific (multiple species) genome comparisons, although it was largely envisioned for the former.

**Fig. 1:**
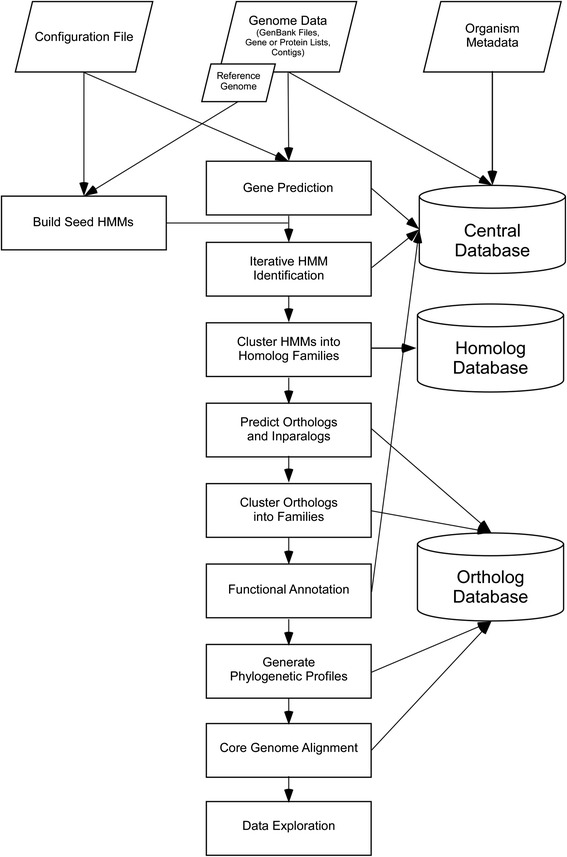
Schematic of the DeNoGAP analysis pipeline. Parallelograms represent input data. Rectangles indicate processes. Cylinders represent databases. The reference genome is used to initiate the construction of HMMs and seed the annotations. While any genome can be used as the reference genome, the use of a well-annotated finished (closed) genome is preferred

A top-level execution script controls the flow of the pipeline by managing the input parameters and calling the modules necessary for executing different analysis phases. Most of the analysis phase except the iterative comparison step can run independently of other phases, provided appropriate parameters and data files are defined in the configuration file given as input to the main execution script. The output(s) from each analysis steps are parsed and stored in a relational SQLite database for result management and post-processing (Fig. 1, Additional file 1: Figure S1, Table 1).

**Table 1:** List of software and databases incorporated in the DeNoGAP pipeline

Program Names	Website	Reference
Gene Prediction		
Glimmer	http://ccb.jhu.edu/software/glimmer/index.shtml	[15]
FragGeneScan	http://omics.informatics.indiana.edu/FragGeneScan/	[18]
Prodigal	https://github.com/hyattpd/Prodigal	[17]
GeneMark	http://opal.biology.gatech.edu/GeneMark/	[16]
Sequence Comparison		
BLAST	ftp://ftp.ncbi.nlm.nih.gov/blast	[22]
HMMER	http://hmmer.org	[27]
Multiple Alignment		
Muscle	http://www.drive5.com/muscle	[29]
Kalign	http://www.ebi.ac.uk/Tools/msa/kalign	[32]
Distance Matrix		
Phylip	http://evolution.gs.washington.edu/phylip	[33]
Clustering		
Markov chain Clustering (MCL)	http://micans.org/mcl	[28]
Sequence manipulation		
EMOSS	http://emboss.sourceforge.net	[20]
Functional Annotation		
InterProScan	https://code.google.com/p/interproscan/	[40]
Annotation Database		
UniprotKB / SwissProt	http://www.uniprot.org	[19]
Pfam	http://pfam.xfam.org	[41]
Gene3D	http://gene3d.biochem.ucl.ac.uk/Gene3D/	[42]
SMART	http://smart.embl-heidelberg.de	[43]
ProDOM	http://prodom.prabi.fr/prodom/current/html/home.php	[44]
FingerPRINTScan	http://www.ebi.ac.uk/Tools/pfa/fingerprintscan/	[45]
PANTHER	http://www.pantherdb.org	[46]
HAMAP	http://hamap.expasy.org	[47]
PIR	http://pir.georgetown.edu	[48]
TIGRFAM	http://www.jcvi.org/cgi-bin/tigrfams/index.cgi	[49]
InterPro	http://www.ebi.ac.uk/interpro/	[50]
MetaCyc	http://metacyc.org	[51]
KEGG	http://www.genome.jp/kegg/	[52]
SignalP	http://www.cbs.dtu.dk/services/SignalP/	[53]
TMHMM	http://www.cbs.dtu.dk/services/TMHMM/	[54]
Phobius	http://phobius.sbc.su.se	[55]
GeneOntology	http://geneontology.org	[56]
SQL Database		
SQLite	https://www.sqlite.org	

### Input data

DeNoGAP take four input parameters from the command line: (1) user-defined table of organism metadata (e.g. time and place of isolation, host, etc.); (2) directory path where SQLite database should be created; (3) name of the SQLite database; and (4) configuration file that defines options for processing input genomic data and performing analysis.

DeNoGAP can process genomic data from multiple formats including: GenBank files, fasta formatted genome sequences (chromosome, plasmid or contig), protein sequences, or coding gene sequences. DeNoGAP parses GenBank files and extracts gene coordinates, functional annotations, and sequence information for the genomes. If the input genomic data is in the form of a multi-fasta formatted genome sequence, DeNoGAP predicts gene coordinates and coding and protein sequences using methods described in “Genomic feature prediction” section.

DeNoGAP requires seeding with one or more reference genomes to identify the initial genomic features and sequences that form the basis for later comparative analyses and functional annotations. Although any genome sequence can act as a seed, we recommend using one or more fully closed and well-annotated genome when possible since annotations carry forward through the analysis. Draft genomes can also be used as seeds when necessary. While these will likely have poorer quality gene predictions and annotations, this will not affect homolog clustering in later steps.

DeNoGAP stores the protein and coding sequences and genomic feature information for all genomes into the SQLite database prior to any downstream analysis. Additional genomes can be added to the analysis at any time. DeNoGAP appends new genomic data into the existing SQLite database and performs iterative comparison of new data with the existing information from previously analyzed genomes. The data is accessible via a basic graphical user interface (GUI).

### Genomic feature prediction

DeNoGAP predicts coding gene sequences from prokaryotic genome sequences using four gene prediction programs: Glimmer, GeneMark, FragGeneScan, and Prodigal [15–18]. Glimmer, GeneMark, and Prodigal use self-trained data to predict genes while FragGeneScan use sequencing error and codon usage models to predict genes in fragmented genome assemblies. The gene prediction results from all four programs are combined and parsed to identify reliable gene candidates. Predicted open read frames (ORFs) are considered reliable if they are recovered by at least two programs, and the longest ORF is selected when the methods disagree. In some cases, gene prediction algorithms predict ORFs that overlap with one another over a few bases. To avoid predicting a large number of genes with overlapping and repeated sequences, DeNoGAP by default considers ORFs with more than 15 bases overlap as a single ORF. The threshold value for the overlap region can be defined by the user in the configuration file.

Gene sequences predicted by only a single program may be the result of algorithm error or bias, and therefore require further verification before including in the compiled set of reliable gene candidates. ORFs predicted by a single program are verified by BLAST against the UniProtKB/SwissProt database [19]. Singleton ORFs (occurring in only one strain) are also verified by comparing the length of the sequence to the user-defined minimum gene length cut-off. We recommend that singleton ORFs should be only included in the set of reliable gene candidate if they satisfy at least one of the two verification criteria. Nucleotide sequences of the predicted coding regions are translated into amino acid sequences using transeq program from EMBOSS software suite [20]. The results from the gene prediction phase are stored in GenBank file format. All features are named according to genome abbreviation and a feature identification number, which are zero-padded sequential numbers unique for each feature (e.g. strain-code_00001).

### Prediction of homolog families and orthologs

**Fig. 2:**
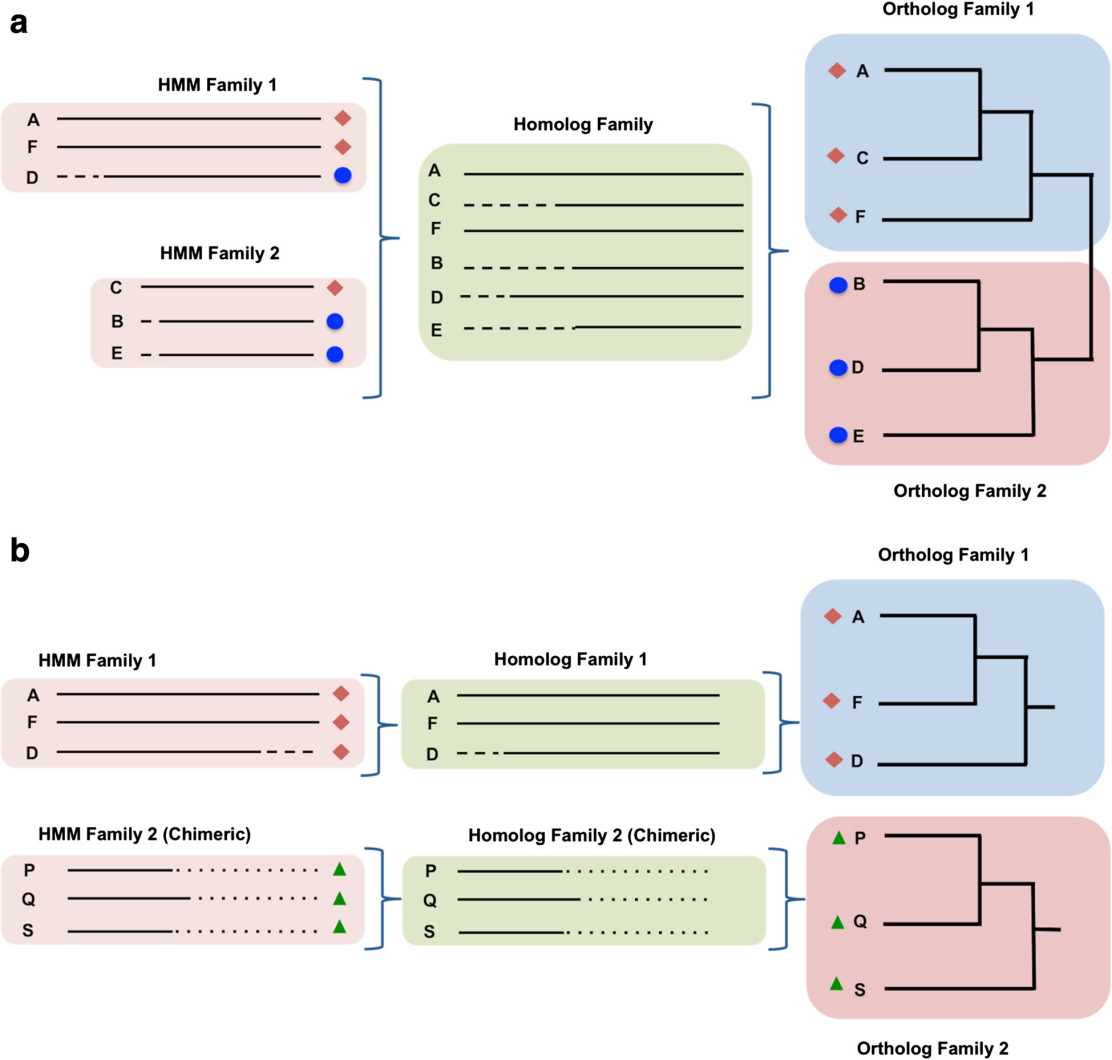
**a** Schematic representation of the relationship between HMM families, homolog families and ortholog families. The diagram shows clustering of related protein sequences (marked with red and blue symbols) into HMM families, homolog family and ortholog families. **b** Schematic representation of the relationship between chimeric protein family (green) and other partially similar protein families (red). The chimeric protein families are clustered separately as new protein family

Homology and orthology prediction are major analysis phases of DeNoGAP as execution of all other analyses is dependent on these results. Profile-sequence alignment is one of the most sensitive methods developed for generating accurate protein alignments [21]. A number of software tools have been developed that implement profile alignment methods for homolog detection from multiple genome sequences [22–24]; however, very few programs are available that use this approach for large-scale comparative genome analysis and ortholog prediction [23, 25, 26]. DeNoGAP develops profile hidden Markov models via HMMER and Markov clustering algorithm (MCL) to iteratively cluster globally similar and highly related protein sequences into HMM families and homolog families respectively [27, 28]. Once homolog families are identified, DeNoGAP predicts ortholog pairs from the families based on reciprocal smallest pairwise genetic distance (Fig. 2a). This step requires a prior designated outgroup in order to minimize false positive ortholog prediction due to the gene loss. Choosing an appropriate outgroup genome is an important factor for reliable ortholog prediction, and is discussed further below. DeNoGAP also predicts chimera-like sequences that are formed through the fusion of portions of one or more gene sequences to produce a new protein. The tool clusters chimeras separately as new protein families, while retaining a link to the related sequences (Fig. 2b). The homology and orthology prediction phases can be divided into five sub-steps as described below.

#### Prediction of seed HMM model families

**Fig. 3:**
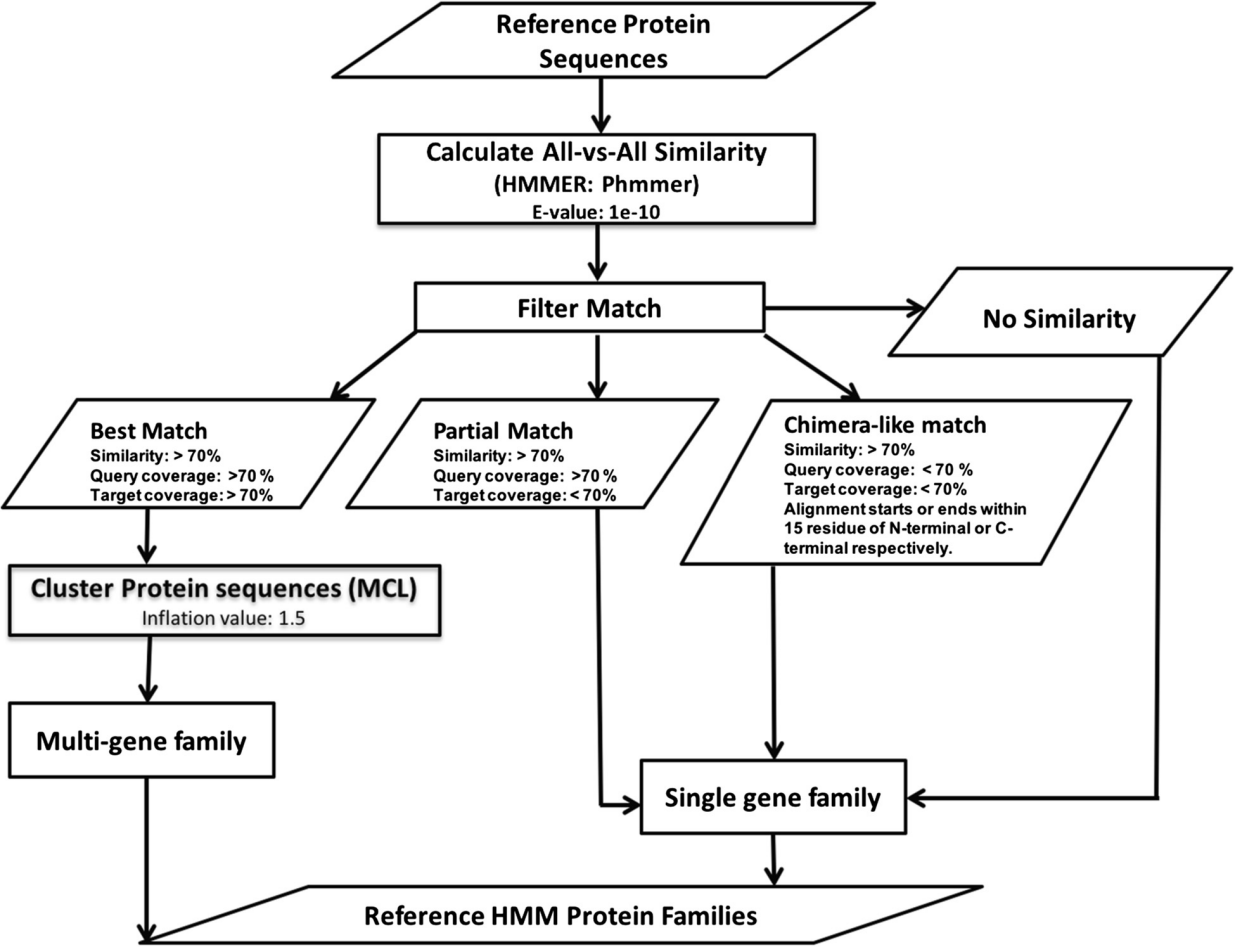
Flowchart of steps for prediction of reference HMM model families

The first step in the ortholog prediction phase is a pairwise comparison of protein sequences extracted from one or more annotated seed genomes to build the initial HMM families. The pipeline uses phmmer program in HMMER package with an E-value threshold of 1e-10 for assessing similarity between each pair of protein sequences (Fig. 3). The pairwise similarity results are parsed to predict pairs of sequences with significant global similarity, partial similarity or no significant similarity to any other protein sequence in the database. A protein pair is predicted as globally significant only if both the query and target sequence have more than 70 % sequence similarity and 70 % sequence coverage. The sequence coverage in the context of DeNoGAP is defined as percentage of the query sequence that overlaps subject sequence and vice versa. If only one of the sequence in a pair has more than 70 % sequence coverage than the query sequence for that pair is identified as being partially similar to the target sequence. The similarity results are also parsed to identify protein sequences having N-terminal or C-terminal ends partially aligning with the N-terminal or the C-terminal of any profile-HMM or singleton sequence respectively. Such protein sequences are considered as potential chimeric-like sequence.

The parsed similarity information is subjected to the MCL algorithm, which clusters significantly similar protein sequences into the protein families. Protein sequences with significant global alignments are grouped together into protein families. Singleton, partial sequences, and chimera-like protein sequences are clustered separately, with each forming a new protein family. We avoid grouping partial and chimera-like sequences with longer similar sequences at this point in the pipeline to prevent errors in construction of the profile-HMM models. These sequences are reconnected later during clustering of profile-HMM models into homolog families.

#### Selection of diverse representative sequence and constructing HMM models

After clustering of protein sequences into globally similar protein families using MCL, each family is subjected to construction of HMM-profile representing that family. Prior to construction of HMM-profile, each protein family is scanned to select diverse representative sequences. The group of diverse representative sequences from each model family is subjected to multiple sequence alignment using MUSCLE [29]. Any sequences that are 100 % identical over the entire length are merged as one sequence for construction of profile-HMM model. This step minimizes the effect of sampling bias in the construction of the HMM.

The pipeline uses hmmalign when aligning new sequences to an existing HMM model. A profile-HMM model is constructed from the protein alignment of each model family using hmmbuild. All profile-HMM models are added to the profile-HMM database and formatted using hmmpress for sequence-profile comparisons. Singleton groups are also added to the singleton sequence database.

#### Iterative prediction of HMM model families in new genome

**Fig. 4:**
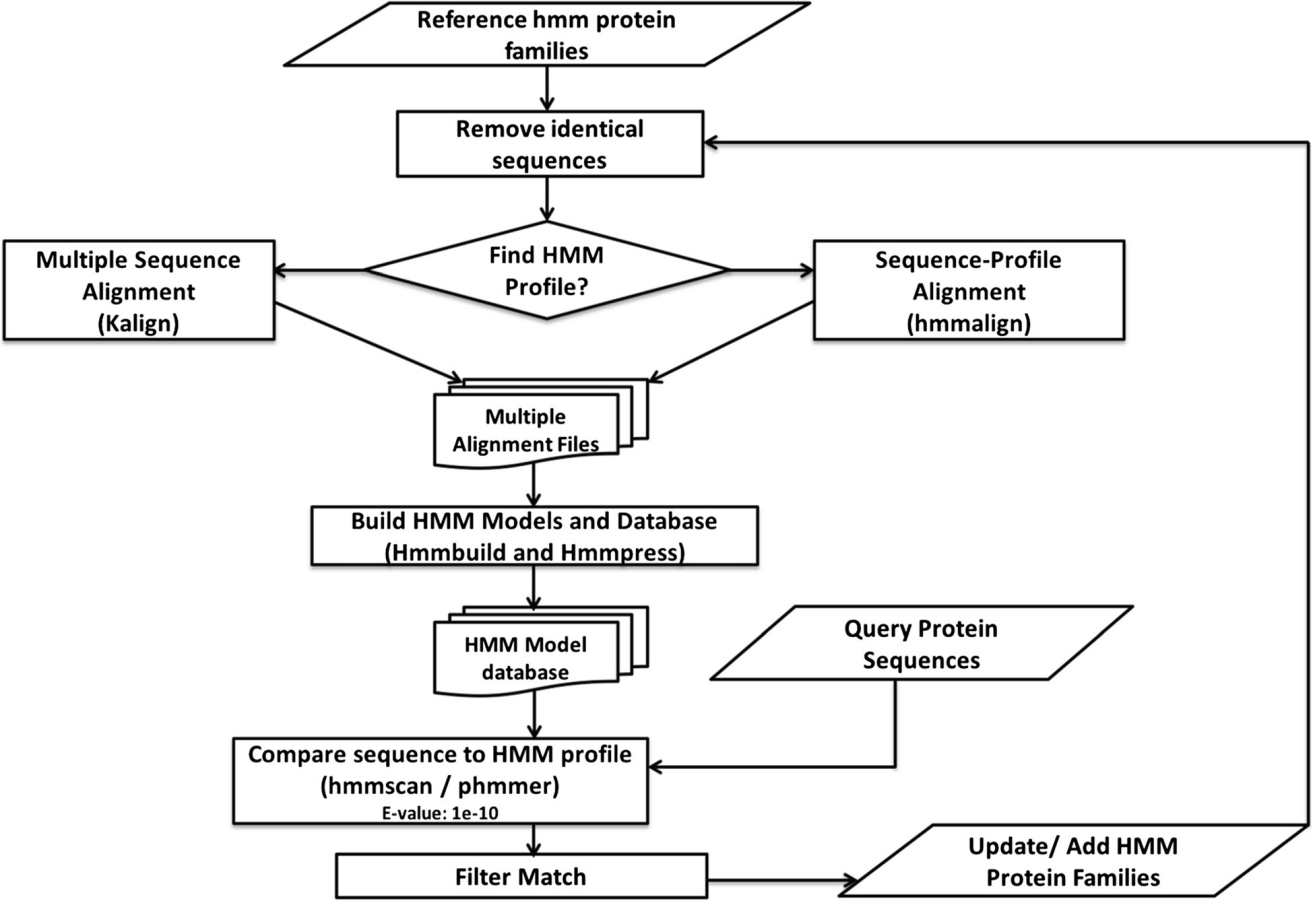
Flowchart of steps for prediction of homolog and novel HMM model families in new genomes using iterative clustering algorithm

In order to predict homologs and novel protein families from a new genome sequence, DeNoGAP iteratively compares protein sequences from the new genome with the existing profile-HMM database and singleton sequence database using hmmscan and phmmer program respectively (Fig. 4). The database size for the comparison is fixed to the size of the model database for consistent E-value calculation. The sequence similarity results are parsed to predict globally similar homologs, partially similar homologs, singletons and chimera-like sequences in the new genome using the same approach as described in earlier step for reference seed family clustering. The globally similar homolog sequences from new genomes are added to the best matching HMM model; whereas, chimera, singleton, and partially similar sequences are clustered as novel families. All steps in iterative clustering phase are repeated for each new genome. During each iteration, DeNoGAP selects diverse sequences from newly predicted homologs and updates and refines the existing HMM models with these new sequences. It also identifies novel families in the new genomes.

#### Clustering of HMM model families into homolog families

Because DeNoGAP is designed to construct HMM models from only globally similar protein sequences; truncated or chimeric-like protein sequences form their own unique model families. As a result of this criteria, there is inflation in the number of predicted HMM model families and a potential loss of information about these relationships. Therefore, after completion of iterative prediction of HMM model families, DeNoGAP identify links between model families where member(s) from one family share significant partial similarity with members of another model family. DeNoGAP does this by identifying pairs of related HMM families from the calculated similarity information such that at least one member of the short family shares partial match with a member of the longer family. The HMM families are clustered using a single-linkage clustering approach via a customized R code in the DeNoGAP. The model families linked with each other are clustered into the larger family; thereby, reestablishing homolog relationships between truncated or chimeric sequences and to their potential parent family.

#### Prediction of ortholog and inparalog pairs

Orthologs are genes that decent from a common ancestor and arise due to speciation or diversification of that ancestor into independent species or strains. In contrast, paralogs are the genes that are related through a duplication event, while inparalogs are paralogous loci which duplicated after a speciation event and are therefore found in the same species [30]. One of the major goals of DeNoGAP is to break down homolog families into ortholog and paralog relationships. While there is no perfect way to accomplish this, we use pairwise smallest reciprocal amino acid distances from one or more outgroup genomes defined a priori by the user to predict orthologous relationship between pairs of protein sequences.

Choosing an appropriate outgroup genome is an important factor for reliable ortholog prediction. The selected outgroup genome(s) should be from a strain or species that is closely enough related to the target strains to have a high likelihood of sharing many homologous sequences, but divergent enough to minimize the likelihood of frequent recombination with these strains. While no rule will work in all cases, selecting distinct species from the same genus is usually a reasonable starting point. It is also possible to use the level of identity at the 16S rRNA locus, as distinct species are typically less than 97 % identical. A more thorough approach would require performing a phylogenetic analysis on a number of loci encoding housekeeping genes, such as is performed in multilocus sequence analysis [31].

**Fig. 5:**
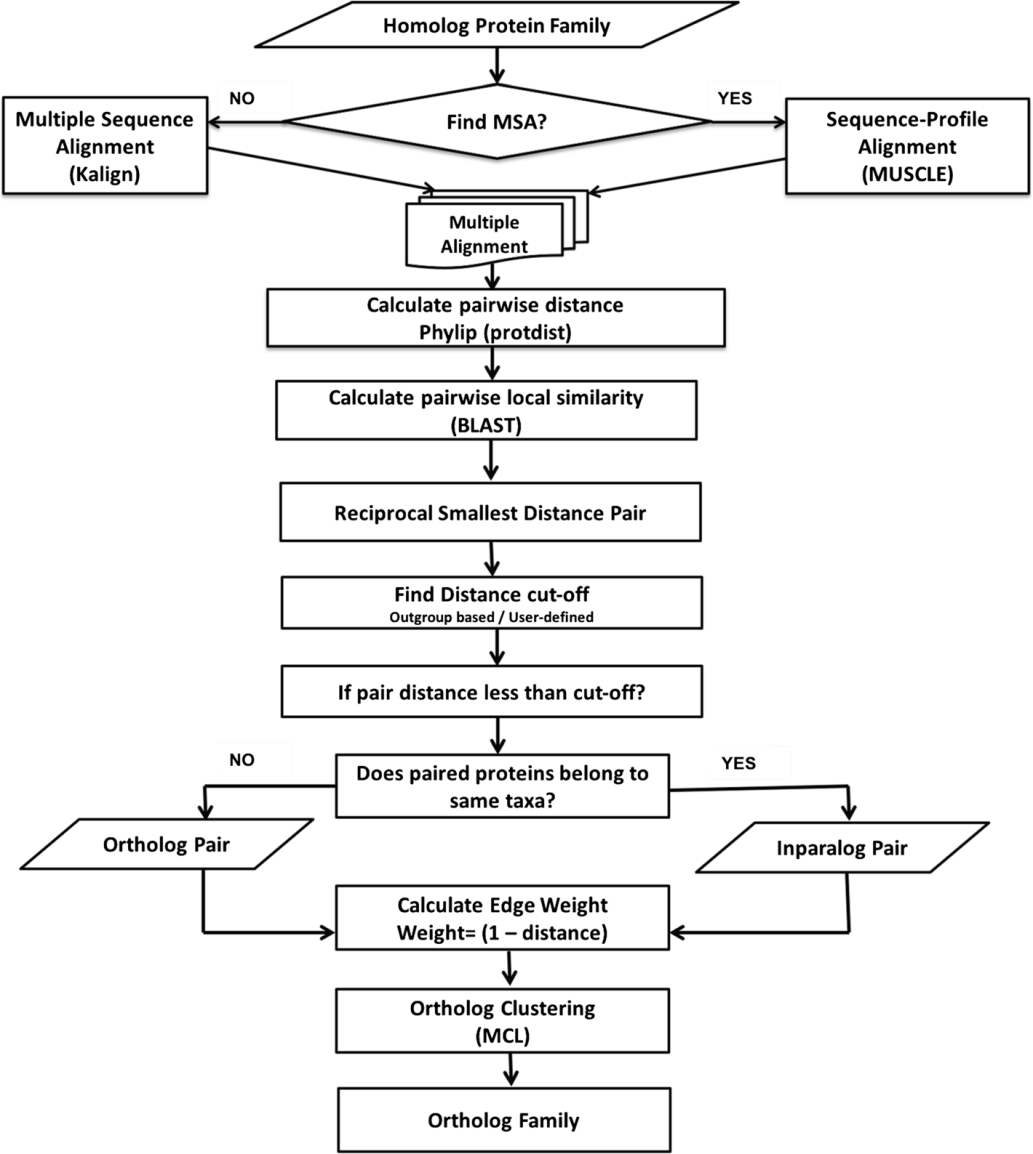
Flowchart of steps for prediction of ortholog, inparalog pairs from homolog families using pairwise protein distance information and clustering pairs into ortholog families using MCL algorithm

DeNoGAP identifies ortholog and inparalog protein sequences from homolog families using a reciprocal minimum amino acid distance approach (Fig. 5). Sequences clustered in each homolog families are aligned using Kalign, and pairwise amino acid distances are calculated using protdist from the Phylip package with the Jones-Taylor-Thoronto (JTT) substitution model [32–34]. Pairwise local sequence identity and sequence coverage between each pair of sequences in each homolog family are calculated using BLASTP [22]. Orthologs and paralogs are distinguished using the standard reciprocal smallest distance logic. For each protein sequence *p*_*A*_ in the genome *A*, the pipeline identifies corresponding protein sequence *q*_*B*_ in the genome *B* that shares smallest reciprocal amino acid distance, and significant local sequence identity and sequence coverage. This reciprocal smallest distance relationship suggests that *p*_*A*_ and *q*_*B*_ are potential orthologs. Unfortunately, this simple relationship can break down under a wide variety of condition, for example when there is differential loss of orthologs. In these case, outgroup sequences can help distinguish orthologs from paralogs. If more than one outgroup sequence is available for a family then the distance cutoff is estimated based on the outgroup protein sequence having the minimum distance from protein under consideration.

**Fig. 6:**
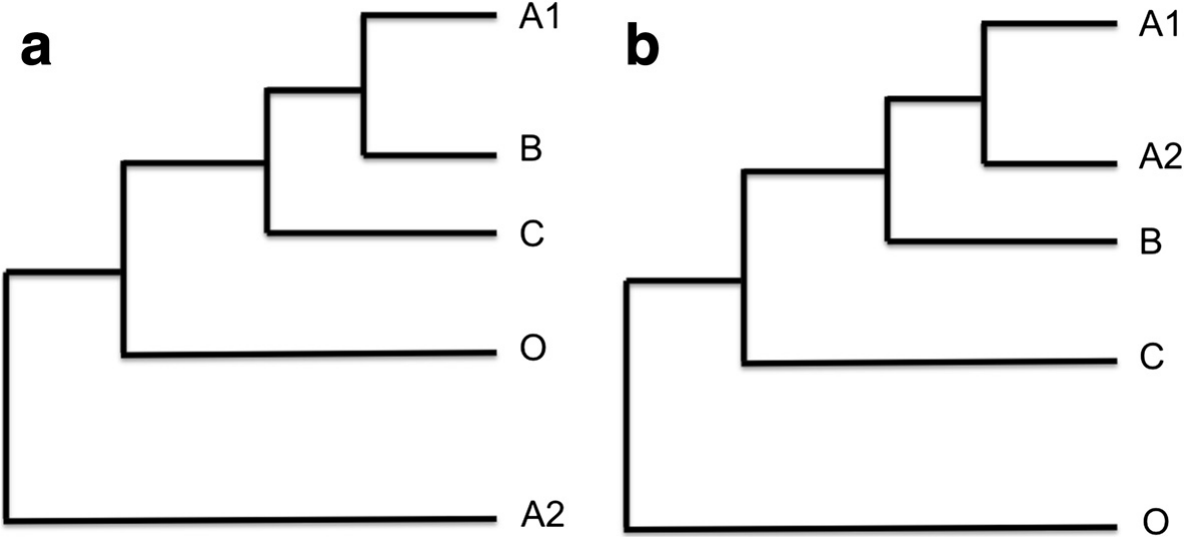
Schematic diagram showing relationships between pair of proteins used for ortholog and inparalog protein prediction by the de-novo pipeline. The outgroup is denoted by taxa O. **a** Ortholog relationship between taxa A, B and C. **b** Inparalog relationship between A1 and A2

If no outgroup is available, DeNoGAP uses a user-defined distance threshold value as a cut-off for distinguishing orthologous and paralogous proteins. Pairs of proteins are predicted to be orthologs if the amino acid distance between two protein sequences is smaller than the distance cut-off (Fig. 6a). In the case of duplication events that occurred after the speciation event, the pipeline identifies pairs of proteins found in the same strain as inparalogs if the distance between the two sequences is equal to or smaller as their individual distances from all the proteins across other genomes (Fig. 6b).

Because orthology is not transitive, DeNoGAP clusters predicted ortholog and inparalog pairs into ortholog families using the MCL algorithm such that each protein sequence in the family shares significant sequence identity with at least one other protein in the family. As shown in Fig. 5, the MCL edge weight for each pair of ortholog and inparalog proteins is calculated by subtracting the pairwise amino acid distance from 1. Although, a more sophisticated weighting scheme can be envisioned, this simple scheme for clustering protein sequences using amino acid distances generates results in good agreement with OrthoMCL (see section on Validation of Ortholog Prediction below).

### Identification of core and variable protein families

Studying gene gain and loss by examining the identity and distribution of core (i.e. those genes present in all strains) and variable genes (i.e. those “accessory” or “dispensable” genes that vary in their distribution among strains) can provide insights into strain evolution, plasticity and environmental adaptation [35, 36]. DeNoGAP generates a binary phylogenetic profile of presence and absence for protein families across all compared genomes based on predicted ortholog information. The phylogenetic profile is a binary matrix denoting the presence and absence of each locus across many genomes [37].

While the core genome is traditionally defined as those genes present in all strains within a defined group, the use of draft genomes can artifactally reduce the size of the core genome if a true core gene is disrupted due to an assembly issue. To compensate for this potential problem DeNoGAP permits the user to define a minimum prevalence threshold (e.g. present in 95 % of strains) for the identification of core genes.

Once a core genome cutoff is defined, the multiple sequence alignment for each core gene is extracted from the alignment stored in the SQLite database. These alignments are then concatenated together to create a core genome alignment, which can be used the construction of a phylogenetic super-tree and downstream comparative analyses [29, 38, 39].

### Functional annotation

DeNoGAP performs functional annotation of protein families by assigning annotations to each protein sequence using InterProScan. The pipeline scans each protein sequence against ten different databases in the InterProScan standalone suite [40]. The annotation resources in the InterProScan suite include InterPro, Pfam, SMART, TIGRFAM, ProDom, PANTHER, PIR, FingerPrintScan, Gene3D, HAMAP, MetaCyc, and KEGG database [41–52]. It also provides prediction of signal peptides and transmembrane domains for each protein sequence using SignalP, TMHMM, and Phobius respectively [53–55]. InterProScan assigns protein sequences with the Gene Ontology (GO) terms associated with Interpro annotation [56].

### Storing and querying analysis results

DeNoGAP use three relational SQL database for managing and post-processing of the output(s) from different analysis phases. The databases are created using SQLite, which is an in-process library that implements a self-contained, server-less and zero-configuration, transactional SQL database engine. The architecture of three SQLite database created by DeNoGAP for storing results is shown in Additional file 2: Figure S2. The central database stores metadata for genomes, sequences, genomic features, functional annotations and sequence-profile similarities from the iterative addition of new genomes. The second database with prefix “HomologDB” stores mapping information for each protein sequence and its respective hmm-model and homolog family group predicted via the iterative clustering of full-length and partial homolog sequences. The third database with prefix “OrthologDB” stores multiple alignments for homolog families, ortholog and inparalog pairs, sequence similarity between each pair of protein sequences in the homolog family, and phylogenetic profiles of presence and absence for ortholog families across compared genomes. The pipeline uses information stored in the database tables for iterative analysis of new genomes and updates the databases by adding newly analyzed information to the central database and creating a new copy of “HomologDB” and “OrthologDB” database.

DeNoGAP also produces a script to create a searchable graphical user interface (GUI) table for genome information stored in the database. The GUI table allows the user to select groups of species for analyzing the pan-genome of selected species. It allows the user to compare presence and absence of ortholog protein families between selected groups of genomes and identify core, flexible or unique families present in different genomes. It also provides an option to fetch, display and edit annotation for each protein sequence from the database.

## Result and discussion

### Performance evaluation

We tested DeNoGAP using a dataset consisted of 140 prokaryotic genomes, including 122 bacteria and 20 archaea strains (Additional file 3). This full dataset was used to evaluate the processing time of DeNoGAP verses OrthoMCL. Subsets of the full dataset were used to evaluate and demonstrate various components of DeNoGAP. For example, we selected five fully sequenced and manually annotated *Pseudomonas* genomes to evaluate the accuracy of the gene predictions module of DeNoGAP. We used 19 well-curated bacterial genomes that are listed as reference proteomes in the Quest for Ortholog database (questfororthologs.org) for benchmarking the ortholog prediction phase of DeNoGAP [57]. Finally, we selected 32 genomes from the genus *Pseudomonas*, including 22 *Pseudomonas syringae*, two *Pseudomonas aeruginosa*, four *Pseudomonas putida*, three *Pseudomonas fluorescens* and one *Pseudomonas entomophila* to illustrate results obtained from the entirety of the analysis pipeline. The 22 *P. syringae* strains were used as in-group strains; while the other *Pseudomonads* were used for outgroup comparisons. *Pseudomonas syringae* pv. tomato strain DC3000 was chosen as a seed reference genome for the all datasets [58]. All archaea strains were used as outgroup genomes for full dataset.

**Fig. 7:**
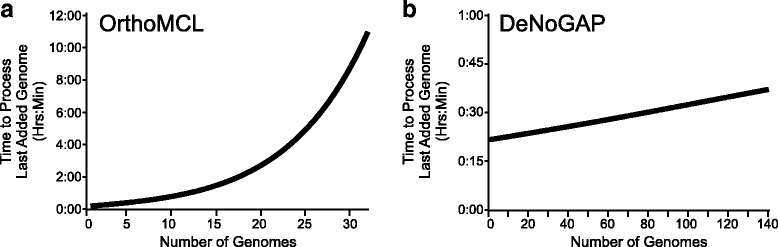
Performance evaluation of the iterative clustering method implemented in the DeNoGAP as compared to reciprocal blast based approach implemented in the OrthoMCL using 140 prokaryotic genomes. Clustering with OrthoMCL was restricted to 32 genomes due to drastic increase in computation time. Each data point on the y-axis represents computation time for the addition of one new genome to an existing dataset. Time-scale of iterative homolog clustering using **a** OrthoMCL or **b** DeNoGAP. Computation time for each iteratively added genome in OrthoMCL increases quadratically with the number of genomes, while computation time for iterative clustering using DeNoGAP increases linearly even after analysis of hundreds of genomes

We evaluated the performance of the iterative clustering strategy implemented in DeNoGAP relative to OrthoMCL by comparing processing time for each successively added genome in our full dataset of 140 strains. The test was performed on a personal computer configured with Linux OS, 2 TB disk space, and 24GB RAM. The comparison of the time-scale between the two approaches showed that the time requirement for processing each new genome via OrthoMCL grows quadratically by O(N^2^), where N is the number of genomes under comparison. In contrast, the time requirement for processing each new genome using DeNoGAP increases linearly based on the increase in the number of predicted novel homolog families (Fig. 7). Since the time to process each new genome only increases with the addition of new homolog families, the iterative addition of genome data to a large number of existing closely related strains have negligible effect on the time since few novel homolog families will be identified. We terminated the OrthoMCL analysis after 32 genomes since the final genome analyzed took approximately 11 h to process. In comparison, the final genome to analyzed from the set of 140 took only approximately 40 min by DeNoGAP. The substantial difference observed in the computation time for OrthoMCL verses DeNoGAP is due to OrthoMCL’s dependence on pairwise analyses of all genomes, and applies to all approaches that rely on pairwise or reciprocal analysis, such as Reciprocal Smallest Distance (RSD) and Ensembl-Compara [11, 12, 64]. The addition of even a single new genome to an existing database using these methods requires the complete pairwise reanalysis of all the genomes in the analysis set. Currently, there is no straightforward way iteratively add new genomes to an existing OrthoMCL database, or identify homolog families for a new genome while updating existing similarity relations. A similar scaling also applies to the disk space and memory requirements for storing and processing output for both the approaches. For example, parsing and analyzing pairwise BLAST results using OrthoMCL requires disk space for the relational database equal to five times the size of the parsed BLAST output file (lge.ibi.unicamp.br/Ortho_MCL_UserGuide.txt). Consequently, this quickly become a limitation when performing pairwise sequence comparisons between hundreds of genomes. In contrast, the iterative clustering algorithm implemented in DeNoGAP stores pairwise similarity information in the form of profile-sequence comparisons, which requires much less disk space due to the condensed representation of multiple sequence alignments inherent in profile-HMMs.

### Validation of gene prediction

DeNoGAP combines output from four microbial gene prediction programs and predicts reliable open reading frames (ORFs) based on overlapping gene region predicted by at least two programs. To validate the accuracy of the gene prediction phase in the DeNoGAP we ran the gene prediction module on five completely sequenced (finished) bacterial genomes from the validation set using two overlap cutoff thresholds (15 and 50 bp) and compared our gene prediction results with the gene annotation information available from GenBank [58–62]. The result shows that DeNoGAP was able to predict ORFs for 94 and 97 % of annotated protein-coding genes (for the 15 and 50 bp thresholds respectively). From these ORFs, 68.2 and 73.1 % had exact start and stop sites to the features described in the GenBank files. Among the genes predicted with incorrect start site, 75.9 and 77.2 % of genes had start codon within 100 nucleotides of the true start site. Approximately 2.5 and 4.2 % of annotated protein-coding genes were not identified by DeNoGAP as reliable ORFs, of which 80 and 88 % overlapped with an adjacent gene beyond the established threshold. Finally, 136 GenBank annotated protein-coding genes were not predicted by any of the four algorithm used by DeNoGAP. Table 2 summarizes the results for gene prediction phase.

**Table 2:** Summary of gene prediction comparison and statistics

Genome Name	Glimmer	GeneMark	Prodigal	FragScan	Combined (15 / 50)	Single (15 / 50)	Total (15 / 50)	Reference Set
PtoDC3000	5836	5944	5862	5950	5659 / 5716	695 / 721	6390 / 6437	5619
PsyB728a	5242	5273	5207	5469	5095 / 5135	662 / 673	5757 / 5808	5089
Pph1448A	5534	5667	5579	5622	5353 / 5416	634 / 661	5987 / 6077	5172
PAO1	5721	5701	5682	8491	4810 / 4927	3740 / 3837	8550 / 8764	5574
Pf01	5659	5798	5738	9045	4869 / 4953	4126 / 4230	8995 / 9183	5722

### Validation of ortholog prediction

**Fig. 8:**
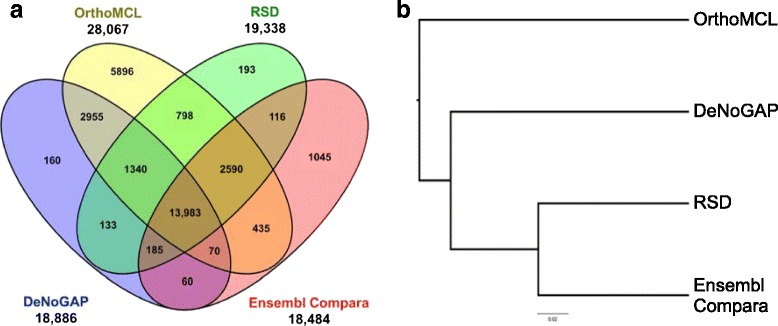
Benchmarking of the ortholog prediction phase for DeNoGAP by comparison of predicted ortholog pairs in a reference proteome from 19 bacterial species derived by the Quest for Ortholog project. **a** Venn diagram showing a comparison of ortholog pairs predicted by DeNoGAP, OrthoMCL, RSD and Ensembl-Compara methods. **b** A cluster analysis of orthologs predicted by the four methods based on differences in their phylogenetic profiles (binary vectors generated for each method indicating the presence or absence of each ortholog in a combined ortholog set)

In order to test the accuracy of DeNoGAP for ortholog prediction, we compared ortholog pairs predicted by DeNoGAP using a well-curated benchmark ortholog dataset of 19 bacterial genomes established as a “reference proteome” by Quest of Ortholog database [63]. The comparison was performed with ortholog pairs calculated using OrthoMCL, RSD and Ensembl-Compara method [11, 12, 64]. We focused on ortholog pairs sharing more than 50 % sequence identity for the benchmark dataset in order to ensure that we had high-confidence in the ortholog calls. The comparison showed that DeNoGAP predicted at least 66.6 % of the total ortholog pairs that were predicted by at least one other method (Fig. 8a), with 46.7 % ortholog pairs predicted by all methods. Only 0.5 % of DeNoGAP’s ortholog predictions were not found by any other approach, while 0.6 %, 3.5 % and 19.7 % of predictions were unique to RSD, Ensembl-Compara and OrthoMCL, respectively. We generated a global set of all orthologs called by the four methods and generated phylogenetic profiles (binary presence/absence vectors) for each approach, which were then subjected to cluster analysis (Fig. 8b). The dendrogram clearly indicates that DeNoGAP performs similarly to the three established analytical approaches.

In order to test the ortholog clustering accuracy of DeNoGAP relative to OrthoMCL, we compared ortholog clusters derived from 195,948 protein sequences from 32 genomes using a granularity parameter (I) of 1.5. DeNoGAP and OrthoMCL clustered protein sequences into 19,914 and 14,377 groups respectively. Of these, 8,703 groups were identical for both methods representing 43.7 % of DeNoGAP groups and 60.5 % of OrthoMCL groups. We also found that 10,204 (70.9 %) of the OrthoMCL groups were a match or subset of DeNoGAP groups, while 18,796 (94.3 %) of the DeNoGAP groups were a match or subset of OrthoMCL groups. We believe that DeNoGAP generates larger numbers of clusters compared to OrthoMCL because it better able to separates highly similar inparalogs into different groups by accounting for gene loss in one or more genomes.

### Prediction of fragmented and chimeric protein families

The algorithm implemented in DeNoGAP for calculating similarity between query sequences and HMM models uses a high alignment coverage cut-off (>70 %) for iterative clustering of globally similar protein sequences. Due to this criterion, protein sequences that exhibit partial similarity with HMM models are clustered initially as new protein families. The analysis of 32 *Pseudomonas* genomes predicted 19,300 protein sequences that had partial similarity with at least one HMM protein family. Approximately, 12,567 (65.1 %) of these sequences displayed significant similarity (query coverage ≥ 70 %) with longer HMM models, suggesting fragmentation of the sequence; whereas, 4,688 (24.2 %) of the sequences showed similarity with HMM models shorter in length. We also found that 1,531 (7.9 %) of protein sequences had significant similarity with both longer and shorter HMM models.

Other than fragmented protein sequences, DeNoGAP also predicts evolutionarily divergent chimera-like protein sequences that are formed through the combination of portions of one or more protein sequences to produce new proteins [65]. The pipeline predicted 514 (2.8 %) of protein sequences had N-terminal or the C-terminal regions with significant similarity to another protein family.

To validate chimera prediction by DeNoGAP, we investigated our results for six known chimeric proteins from *P. syringae* described in the literature. On searching, it was found that DeNoGAP correctly identify four out of six known chimeric proteins. Two of the identified chimera proteins, HopK1, and HopD1 are type III secreted effector protein present in *P. syringae* strain PtoDC3000. The pipeline identified partial similarity with the N-terminus of the type III effector HopAQ1 and HopD2 (also known as HopAO1), respectively [65]. The other two predicted chimeric proteins were the type III effector proteins HopBB1 and HopAE1 in the strain PavBPIC631 with N-terminal similarity to HopF2 and HopW1, respectively [66]. These results suggest that DeNoGAP can efficiently be used for predicting novel chimera proteins as well as families of known chimera proteins in new genomes. However, the currently implemented method for chimera prediction also identifies proteins sharing common domains with multi-domain proteins; therefore, the pipeline can over-estimate the number of chimeric proteins in the genome. Consequently, we recommend that chimeric proteins undergo manual verification.

### Clustering of HMM families into homolog families

**Fig. 9:**
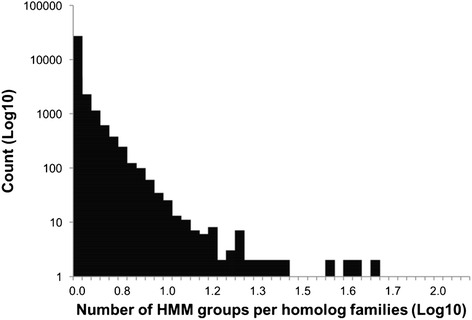
Clustering of HMM protein families related by at least one sequence. The x-axis represents homolog family size in terms of the number of HMM groups clustered in each family

Draft genomes present significant challenges for homolog prediction due to the presence of fragmented proteins [67]. In order to build accurate models for homolog families, DeNoGAP clusters putative fragmented and chimeric proteins into unique families. However, it is important to understand how these families are related to other (e.g. full-length) families due to significant local similarity. DeNoGAP does this via single-linkage clustering of related HMM families. To assess this, we represented the HMM families as nodes (51,166) connected by edges if they shared significant similarity (221,068 edges). We found that the network of related HMM families consisted of total 33,499 homolog family clusters. Out of all the homolog family clusters, only 5,851 (17.46 %) contained two or more HMM families. The other 27,648 (82.53 %) clusters comprised of only one HMM family, suggesting that they were well partitioned and do not share similarity with other HMM families (Fig. 9).

### Identification of core and variable protein families

**Fig. 10:**
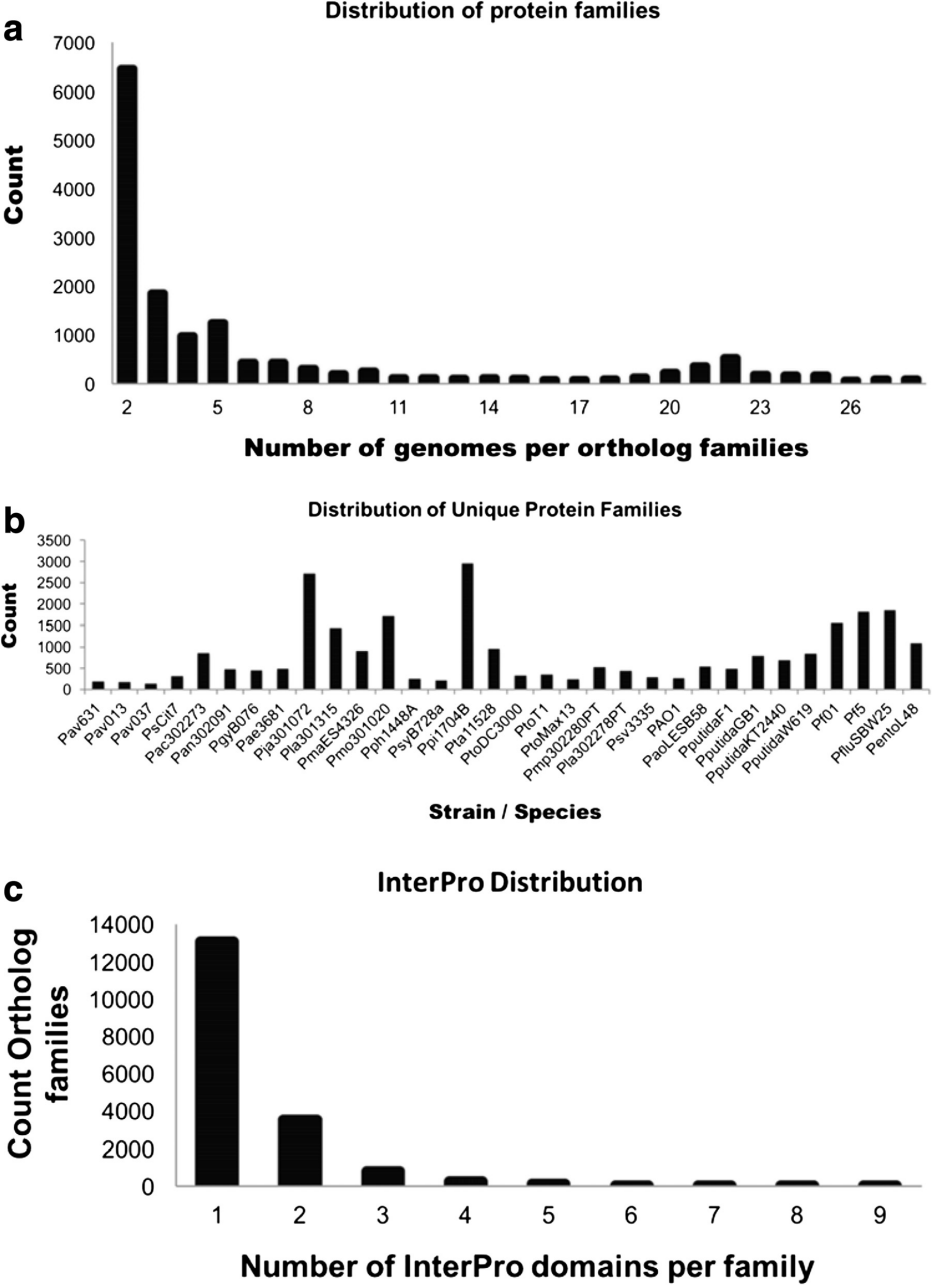
**a** Distribution of genomes in ortholog protein families. The x-axis plots the size of each ortholog families in terms of the number of genomes carry each family. **b** Distribution of lineage-specific (singleton) ortholog families predicted across 32 genomes. **c** Distribution of InterPro annotation across ortholog protein families

In order to analyze the pattern of gene gain and loss, we constructed a phylogenetic profile representing the presence or absence of 16,742 predicted ortholog protein families across the 32 genomes from the phase validation dataset via the profile module in DeNoGAP. For this analysis we defined the core ortholog families as those present in at least 90 % of genomes to account for false negative gene predictions resulting from incomplete assemblies of draft genomes. The analysis predicted 1834 (10.95 %) ortholog families as core. The majority of the ortholog families were present in only few genomes, with approximately 62 % of the families present in less than five genomes, while only ~26 % of variable ortholog families were distributed in the mid-range between 5 to 28 genomes (Fig. 10a). We found 25,886 lineage-specific families that consisted of sequences of a single strain (Fig. 10b). Most strains had 100 to 500 lineage-specific families with an exception in Pla301315, Ppi1704B, Pmo301020 and Pja301072, where the pipeline predicted more than 1000 lineage-specific families. In the case of Pla301315 strain, a significant number of lineage-specific families are reported due to the presence of mega-plasmid of over 1 MB in size [68]. It is not clear why the other strains have so many lineage-specific genes. While the presence of plasmids may account for some, it is likely that many are due to assembly artifacts [67].

DeNoGAP produces a concatenated MUSCLE-based multiple sequence alignment from all core protein families [29]. The core genome alignment can be used as input to an external tree-building program for creating a core genome super-tree for inferring clonal phylogenetic relationship among strains [37, 38].

### Functional annotation

We functionally annotated each protein family predicted for 32 phase validation genomes by assigning Interpro annotation to the families using annotation module in DeNoGAP. The analysis identified 11,364 (67 %) ortholog families and 6,423 (25 %) lineage-specific families with one or more Interpro annotation (Fig. 10c). The remaining families had no functional annotation. These results are consistent with supposition that many lineage-specific families are assembly artifacts. The list of highly enriched Interpro annotations and their frequency in predicted ortholog families is given in (Additional file 4).

### Exploration and visualization of genomic data

DeNoGAP includes scripts for creating a local web-based database explorer that reads the three SQLite databases and builds a query platform for exploration and visualization of genomic information. The query platform allows users to select a subset of genomes from the database for comparison of core, flexible and unique protein families (Additional file 5: Figure S3) [35]. It provides users with an option to set thresholds for defining core protein families to account for missed genes due to assembly errors. It also permits annotation-specific searches. The program retrieves protein IDs and their associated annotation information based the search query, and outputs the results in an HTML table. The user can further select individual feature IDs to visualize genomic information and annotations for each gene/protein sequence.

## Conclusion

DeNoGAP provides a complete package integrating many bioinformatics tools for the analysis of large comparative genomic datasets. The pipeline offers tools and algorithms for the annotation and analysis of both complete and draft genome sequences, and performs analysis tasks including: gene prediction, ortholog prediction, chimera prediction, functional annotation and pan-genome analysis. The modular design of the pipeline makes it relative easy to add new analysis functionalities to the toolkit. One of the major goals while designing DeNoGAP was to provide an integrated and automated workflow for large-scale comparative genomics projects involving hundreds of sequenced genomes; therefore, we have focused on automating the execution of necessary analysis modules, parsing and formatting of output from each analysis phase, and preparing input for the subsequent phase.

While the next-generation sequencing revolution has tremendously increased the number of available genomes for large-scale comparative genomics projects, the computational infrastructure needed for these analyses is often limited. We have designed the DeNoGAP pipeline with the goal of making a sophisticated pipeline that can run on nearly any system with reasonable processing power, memory and disk space, and which easily scales for hundreds of genome. DeNoGAP provides a streamlined workflow to rapidly analyze and annotate newly sequenced and assembled genomes in an iterative manner, and creates a new, or updates an existing, SQLite database. Finally, DeNoGAP provides a database exploration tool that allows researchers to parse and explore the analyzed information for the generation of new hypothesis.

## Availability and requirement

**Project name:** De-Novo Genome Analysis Pipeline (DeNoGAP)

**Project home page:**https://sourceforge.net/projects/denogap/

**Operating system:** Unix, Linux (Ubuntu 12.04 LTS) or higher.

**Programming Language:** Perl

**Other Requirements:** Apache 2 or higher.

**License:** GPL

## Additional files

Additional file 1: Architecture of the DeNoGAP genomics pipeline. The input phase shows the information required at the command line while executing the pipeline. The analysis phase shows various analyses that can be performed using DeNoGAP pipeline. Each analysis can be performed independently of other steps provided required parameters are defined in the respective configuration file of the analysis. (PDF 1372 kb) Additional file 2: Architecture of SQLite databases for DeNoGAP pipeline. (a) Central database: It includes tables to store basic genomic information, sequences, functional annotations predicted using InterProScan, and sequence-profile similarity information. (b) HomologDB: It includes tables to store list of HMM family pairs that are linked by at least one significantly similar partial sequence, and mapping information for each protein sequence on its respective HMM family and Homolog family. (c) OrthologDB: It includes tables to store pairwise distance information for pairs of ortholog and inparalog, pairwise local similarity information between each pair of protein in the family, homolog multiple alignment and protein family presence and absence information as binary matrix and tabular list. (PDF 996 kb) Additional file 3: List of genomes used for development and testing of the DeNoGAP pipeline. (XLSX 25 kb) Additional file 4: List of predicted InterPro annotation and their frequency in predicted ortholog families. (XLSX 125 kb) Additional file 5: Graphical interface for exploring data generated using DeNoGAP pipeline. (a) Main page of GUI for selection of genomes and setting parameters for comparison. (b) Display of gene list as a result of protein family profile comparison between selected genomes. (c) Display of detailed information for individual protein or genes. (PDF 6662 kb)
