# Supplementary material for: A De-Novo Genome Analysis Pipeline (DeNoGAP) for large-scale comparative prokaryotic genomics studies
Source: BMC Bioinformatics. 2016 Jun 30;17:260. doi: 10.1186/s12859-016-1142-2 (PMC4929753; doi:10.1186/s12859-016-1142-2)
Supplement: Additional file 1: — Architecture of the DeNoGAP genomics pipeline. The input phase shows the information required at the command line while executing the pipeline. The analysis phase shows various analyses that can be performed using DeNoGAP pipeline. Each analysis can be performed independently of other steps provided required parameters are defined in the respective configuration file of the analysis. (PDF 1372 kb) [file 12859_2016_1142_MOESM1_ESM.pdf]

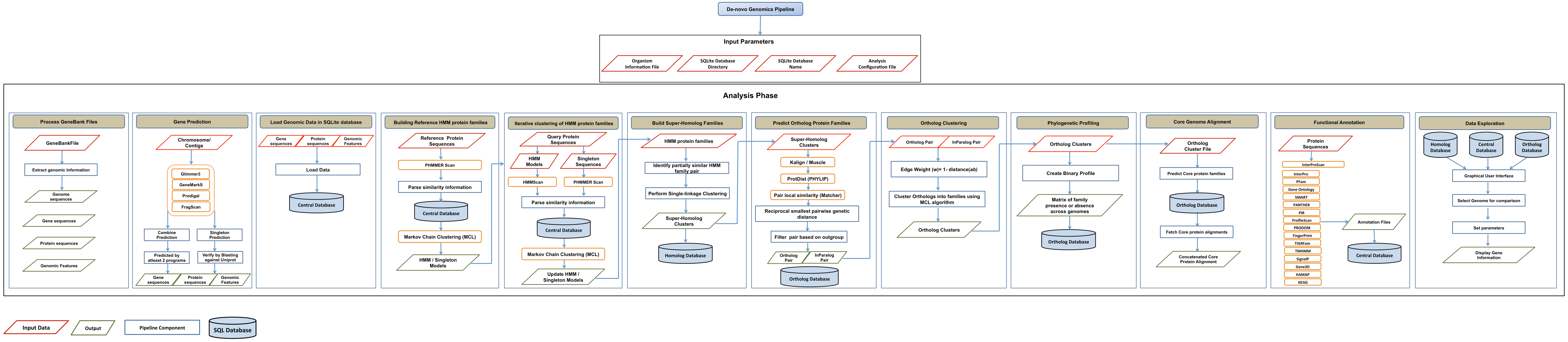

De-novo Genomics Pipeline
Input Parameters
Organism Information File
SQLite Database Directory
SQLite Database Name
Analysis Configuration File
Analysis Phase
Process GeneBank Files
GeneBankFile
Extract genomic Information
Genome sequences
Gene sequences
Protein sequences
Genomic Features
Gene Prediction
Chromosome/ Contigs
Glimmer3
GeneMarkS
Prodigal
FragScan
Combine Prediction
Singleton Prediction
Predicted by atleast 2 programs
Verify by Blasting against Uniprot
Gene sequences
Protein sequences
Genomic Features
Load Genomic Data in SQLite database
Gene sequences
Protein sequences
Genomic Features
Load Data
Central Database
Building Reference HMM protein families
Reference Protein Sequences
PHMMER Scan
Parse similarity information
Central Database
Markov Chain Clustering (MCL)
HMM / Singleton Models
Iterative clustering of HMM protein families
Query Protein Sequences
HMM Models
Singleton Sequences
HMMScan
PHMMER Scan
Parse similarity information
Central Database
Markov Chain Clustering (MCL)
Update HMM / Singleton Models
Build Super-Homolog Families
HMM protein families
Identify partially similar HMM family pair
Perform Single-linkage Clustering
Super-Homolog Clusters
Homolog Database
Predict Ortholog Protein Families
Super-Homolog Clusters
Kalign / Muscle
ProtDist (PHYLIP)
Pair local similarity (Matcher)
Reciprocal smallest pairwise genetic distance
Filter pair based on outgroup
Ortholog Pair
InParalog Pair
Ortholog Database
Ortholog Clustering
Ortholog Pair
InParalog Pair
Edge Weight (w)= 1- distance(ab)
Cluster Orthologs into families using MCL algorithm
Ortholog Clusters
Phylogenetic Profiling
Ortholog Clusters
Create Binary Profile
Matrix of family presence or absence across genomes
Ortholog Database
Core Genome Alignment
Ortholog Cluster File
Predict Core protein families
Ortholog Database
Fetch Core protein alignments
Concatenated Core Protein Alignment
Functional Annotation
Protein Sequences
InterProScan
InterPro
PFam
Gene Ontology
SMART
PANTHER
PIR
ProfileScan
PRODOM
FingerPrint
TIGRFam
TMHMM
SignalP
Gene3D
HAMAP
KEGG
Annotation Files
Central Database
Data Exploration
Homolog Database
Central Database
Ortholog Database
Graphical User Interface
Select Genome for comparison
Set parameters
Display Gene Information
Input Data
Output
Pipeline Component
SQL Database

Program Names	Website	Reference
**Gene Prediction**		
Glimmer	http://ccb.jhu.edu/software/glimmer	[1]
FragScan	http://omics.informatics.indiana.edu/mg/get.php	[2]
Prodigal	http://prodigal.googlecode.com	[3]
GeneMark	http://opal.biology.gatech.edu	[4]
**Sequence Comparision**		
BLAST	ftp://ftp.ncbi.nlm.nih.gov/blast	[8]
HMMER	http://selab.janelia.org/software/hmmer3	[13]
**Multiple Alignment**		
Muscle	http://www.drive5.com/muscle	[15]
Kalign	http://msa.sbc.su.se/downloads/kalign	[16]
**Distance Matrix**		
Phylip	http://evolution.gs.washington.edu/phylip	[17]
**Clustering**		
Markov chain Clustering (MCL)	http://micans.org/mcl	[14]
**Sequence manipulation**		
EMOSS	http://emboss.sourceforge.net	[6]
**Functional Annotation**		
InterProScan	https://code.google.com/p/interproscan/	[29]
**Annotation Database**		
UniprotKB / SwissProt	http://www.uniprot.org	[5]
Pfam	http://pfam.xfam.org	[30]
Gene3D	http://gene3d.biochem.ucl.ac.uk/Gene3D/	[31]
SMART	http://smart.embl-heidelberg.de	[32]
ProDOM	http://prodom.prabi.fr/prodom/current/html/home.php	[33]
FingerPRINTScan	http://www.ebi.ac.uk/Tools/pfa/fingerprintscan/	[34]
PANTHER	http://www.pantherdb.org	[35]
HAMAP	http://hamap.expasy.org	[36]
PIR	http://pir.georgetown.edu	[37]
TIGRFAM	http://www.jcvi.org/cgi-bin/tigrfams/index.cgi	[38]
InterPro	http://www.ebi.ac.uk/interpro/	[39]
SignalP	http://www.cbs.dtu.dk/services/SignalP/	[40]
TMHMM	http://www.cbs.dtu.dk/services/TMHMM/	[41]
GeneOntology	http://geneontology.org	[42]
**SQL Database**		
SQLite	https://www.sqlite.org	
