# Supplementary figures and images for: A De-Novo Genome Analysis Pipeline (DeNoGAP) for large-scale comparative prokaryotic genomics studies
Source: BMC Bioinformatics. 2016 Jun 30;17:260. doi: 10.1186/s12859-016-1142-2 (PMC4929753; doi:10.1186/s12859-016-1142-2)

(a)

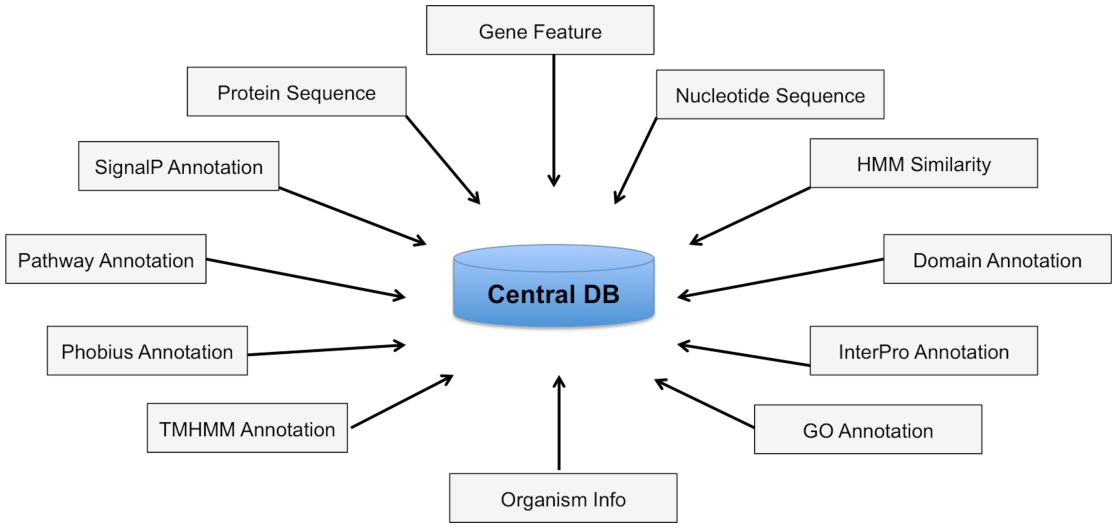

(b)

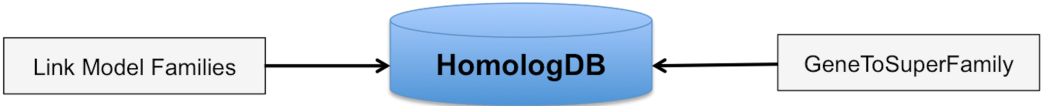

(c)

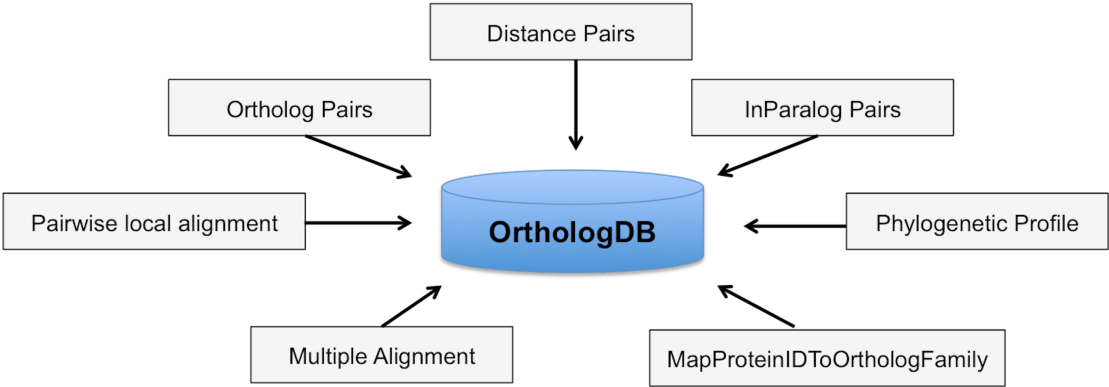

Supplement: Additional file 2: — Architecture of SQLite databases for DeNoGAP pipeline. (a) Central database: It includes tables to store basic genomic information, sequences, functional annotations predicted using InterProScan, and sequence-profile similarity information. (b) HomologDB: It includes tables to store list of HMM family pairs that are linked by at least one significantly similar partial sequence, and mapping information for each protein sequence on its respective HMM family and Homolog family. (c) OrthologDB: It includes tables to store pairwise distance information for pairs of ortholog and inparalog, pairwise local similarity information between each pair of protein in the family, homolog multiple alignment and protein family presence and absence information as binary matrix and tabular list. (PDF 996 kb) [file 12859_2016_1142_MOESM2_ESM.pdf]
