# Supplementary material for: A De-Novo Genome Analysis Pipeline (DeNoGAP) for large-scale comparative prokaryotic genomics studies
Source: BMC Bioinformatics. 2016 Jun 30;17:260. doi: 10.1186/s12859-016-1142-2 (PMC4929753; doi:10.1186/s12859-016-1142-2)
Supplement: Additional file 5: — Graphical interface for exploring data generated using DeNoGAP pipeline. (a) Main page of GUI for selection of genomes and setting parameters for comparison. (b) Display of gene list as a result of protein family profile comparison between selected genomes. (c) Display of detailed information for individual protein or genes. (PDF 6662 kb) [file 12859_2016_1142_MOESM5_ESM.pdf]

(a)

Analysis Report of Test_Set

Find in selected genomes:
- Core Genes
- Variable Genes
- Unique Genes

Define core gene as present in % of genomes: 100
Show result sorted by: Gene Family ID
Search for gene description :

Submit Reset

Show 10 entries — Search: Pseudomonas syring

With Homolog	Without Homolog	genome_name	species	species_type	abbreviation
☐	☐	**Pseudomonas syringae pv. tomato T1**	**Pseudomonas syringae**	**bacteria**	**PtoT1**
☐	☐	**Pseudomonas syringae pv. tomato Max13**	**Pseudomonas syringae**	**bacteria**	**PtoMax13**
☑	☐	**Pseudomonas syringae pv. tomato DC3000**	**Pseudomonas syringae**	**bacteria**	**PtoDC3000**
☐	☐	**Pseudomonas syringae pv. tabaci str. ATCC 11528**	**Pseudomonas syringae**	**bacteria**	**Pta11528**
☑	☐	**Pseudomonas syringae pv. syringae B728a**	**Pseudomonas syringae**	**bacteria**	**PsyB728a**
☐	☐	**Pseudomonas syringae pv. pisi 1704B**	**Pseudomonas syringae**	**bacteria**	**Ppi1704B**
☐	☐	**Pseudomonas syringae pv. phaseolicola 1448A**	**Pseudomonas syringae**	**bacteria**	**Pph1448A**
☐	☐	**Pseudomonas syringae pv. oryzae 36_1**	**Pseudomonas syringae**	**bacteria**	**Por36_1**
☐	☐	**Pseudomonas syringae pv. morsprunorum str. M302280**	**Pseudomonas syringae**	**bacteria**	**Pmp302280PT**
☐	☐	**Pseudomonas syringae pv. mori str. 301020**	**Pseudomonas syringae**	**bacteria**	**Pmo301020**

Showing 1 to 10 of 24 entries (filtered from 142 total entries) — Previous 1 2 3 Next

(b)

Search Result

Genome Name: Pseudomonas syringae pv. syringae B728a — Show Gene Information

Show 10 entries — Search: Copy CSV Excel

Group ID	Homolog Group ID	HMM Group ID	Gene ID	Genome Name	Gene Description	Details
Cluster_10212.1	Cluster_10212	Group2510	Q4ZL22	Pseudomonas syringae pv. syringae B728a	ATP synthase subunit delta	○
Cluster_10520.1	Cluster_10520	Group5462	Q4ZMP2	Pseudomonas syringae pv. syringae B728a	Elongation factor Tu	○
Cluster_10870.1	Cluster_10870	Group4888	Q4ZMN2	Pseudomonas syringae pv. syringae B728a	Transcription termination/antitermination protein NusG	○
Cluster_11008.1	Cluster_11008	Group3165	Q4ZRH5	Pseudomonas syringae pv. syringae B728a	6-carboxy-5,6,7,8-tetrahydropterin synthase	○
Cluster_11077.1	Cluster_11077	Group5954	Q4ZZW4	Pseudomonas syringae pv. syringae B728a	D-amino acid dehydrogenase	○
Cluster_11238.1	Cluster_11238	Group4397	Q4ZRK4	Pseudomonas syringae pv. syringae B728a	Isocitrate dehydrogenase NADP-dependent, monomeric type	○
Cluster_11536.1	Cluster_11536	Group3052	Q4ZM01	Pseudomonas syringae pv. syringae B728a	S-adenosylmethionine synthase	○
Cluster_1198.1	Cluster_1198	Group4047	Q4ZZ83	Pseudomonas syringae pv. syringae B728a	Dihydroxy-acid dehydratase	○
Cluster_1254.1	Cluster_1254	Group1221	Q4ZQB3	Pseudomonas syringae pv. syringae B728a	Unannotated protein sequence	○
Cluster_12972.1	Cluster_12972	Group2410	Q4ZUP4	Pseudomonas syringae pv. syringae B728a	Unannotated protein sequence	○

Showing 1 to 10 of 70 entries — Previous 1 2 3 4 5 6 7 Next

(c)

Edit

GENE : Q4ZL22

Genomic Feature:	
Locus Tag:	Q4ZL22
Protein Id:	Psyr_5123
External database Id:	YP_238188.1
Species / Strain Name:	Pseudomonas syringae pv. syringae B728a
Species Type:	bacteria
Species Abbreviation:	PsyB728a
Genome ID:	NC_007005.1
Genome Type:	Chromosome
Genome Length:	6093698
Index on Genome:	5249
Genomic Location:	6081466 : 6082002 (-1)
Gene Name:	Not available
Feature Type:	CDS
Protein Length:	179
Nucleotide Length:	537
Product Description:	ATP synthase subunit delta
Comments:	Produces ATP from ADP in the presence of a proton gradient across the membrane. The alpha chain is a regulatory subunit.
**Comparative Genomics Information:**	
Homolog Group:	Cluster_10212
Ortholog Group:	Cluster_10212.1
HMM Model Group:	Group2510
**Annotation:**	

GO Annotation:

GO ID	GO Category	GO Description
GO:0005886	Cellular Component	The membrane surrounding a cell that separates the cell from its external environment. It consists of a phospholipid bilayer and associated proteins.

PFam:

PFam ID	Pfam Name	Description	Start	End	Significance
PF00006	ATP-synt_ab	ATP synthase alpha/beta family, nucleotide-binding domain	150	376	1e-10

InterPro:

InterPro ID	Description
IPR020003	ATPase, alpha/beta subunit, nucleotide-binding domain, active site

(d)

EDIT GENE ANNOTATION

**Locus Tag :**	Q4ZL22
**Protein Id :**	Psyr_5123
**External database Id :**	YP_238188.1
**Species / Strain Name :**	Pseudomonas syringae pv. syringae B728a
**Species Abbreviation :**	PsyB728a
**Genome ID :**	NC_007005.1
**Genome Type :**	Chromosome
**Genome Length :**	6093698
**Index on Genome :**	5249
**Feature Start :**	6081466
**Feature End :**	6082002
**Strand :**	+ / -
**Frame :**	1
**Gene Name :**	Not available
**Feature Type :**	CDS
**Protein Length :**	179
**Nucleotide Length :**	537
**Product Description :**	ATP synthase subunit delta
**Homolog Group :**	Cluster_10212
**Ortholog Group :**	Cluster_10212.1
**HMM Group :**	Group2510

GO Id : GO:0005886 **GO Category :** Cellular Component **GO Description :** The membrane surrounding a cell that separates the cell from its external environment

Pfam Id : PF00006 **Pfam Name :** ATP-synt_ab **Pfam Description :** ATP synthase alpha/beta family, nucleotide-binding domain

Pfam Start : 150 **Pfam End :** 376 **Significance :** 1e-10

InterPro Id : IPR020003 **InterPro Description :** ATPase, alpha/beta subunit, nucleotide-binding domain, active site

Pathway Id : ko00190 **Pathway Description :** Oxidative phosphorylation
